# Parkinson's Disease and Autophagy

**DOI:** 10.1155/2012/429524

**Published:** 2012-10-17

**Authors:** Ana María Sánchez-Pérez, Berta Claramonte-Clausell, Juan Vicente Sánchez-Andrés, María Trinidad Herrero

**Affiliations:** ^1^Clinical and Experimental Neuroscience (NiCE-CIBERNED), School of Medicine, Jaume I University, 12071 Castelló de la Plana, Spain; ^2^Department of Medicine, School of Health Sciences, Jaume I University, Campus Riu Sec, 12071 Castelló de la Plana, Spain; ^3^Department of Neurology, Hospital General Universitari, 12071 Castelló de la Plana, Spain

## Abstract

It is generally accepted that a correlation between neurodegenerative disease and protein aggregation in the brain exists; however, a causal relationship has not been elucidated. In neurons, failure of autophagy may result in the accumulation of aggregate-prone proteins and subsequent neurodegeneration. Thus, pharmacological induction of autophagy to enhance the clearance of intracytoplasmic aggregate-prone proteins has been considered as a therapeutic strategy to ameliorate pathology in cell and animal models of neurodegenerative disorders. However, autophagy has also been found to be a factor in the onset of these diseases, which raises the question of whether autophagy induction is an effective therapeutic strategy, or, on the contrary, can result in cell death. In this paper, we will first describe the autophagic machinery, and we will consider the literature to discuss the neuroprotective effects of autophagy.

## 1. Introduction

Autophagy was initially reported more than 40 years ago [1]. It is a physiological process by which cells remove damaged proteins and organelles through lysosomal degradation. This system prevents the accumulation of products that are not only useless, but potentially toxic. In neurons, this process is considered particularly important since neurons do not replicate; therefore, eventual damaging proteins will not be diluted in subsequent divisions. Autophagy is distinctly regulated in neuronal and nonneuronal cells [2, 3], and recent studies have linked autophagic pathways to several pathological conditions ranging from cancer to neurodegenerative disorders [3, 4]. Moreover, impairment of basal autophagy results in neuronal death [5, 6]. Interestingly, accumulation of proteins is a common feature in several neurodegenerative diseases such as Alzheimer's disease (AD), Parkinson's disease (PD), and Huntington's disease (HD). In AD, hyperphosphorylated tau-containing neurofibrillar tangles and A*β* deposits are found; in PD, aggregated *α*-synuclein is a major component in the Lewy bodies; in HD, N-terminal fragments of mutant huntingtin protein (Htt) are found in intracellular inclusion bodies. These findings led to hypothesize that alterations in the autophagic process were responsible for the aggregation of these toxic proteins and consequently to the onset of disease. According to this idea, several reports document an amelioration of toxicity with removal of accumulation of aggregates (for review see [7]). However, other reports challenge this view and suggest that aggregation of toxic products is not correlated with the degree of neurodegeneration; therefore, protein aggregates are considered an epiphenomenon of the disease, not an underlying factor [8–10]. The literature provides enough evidence to feed controversy; in this paper, we will review the data related to the effects of autophagy on neuroprotection, in particular in connection with PD.

## 2. Autophagy Classification

**Figure 1:**
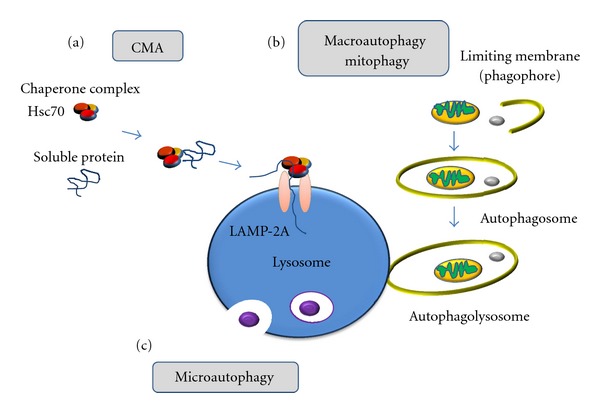
Schematic depiction of the three types of autophagy. (a) Chaperone-mediated autophagy. The cytosolic chaperone protein HSC 70 binds to the substrate protein; the consensus sequence LysPheGluArgGln of the substrate-chaperone complex is recognized by LAMP-2A, a lysosomal membrane receptor. The protein substrate is then unfolded and translocated across the lysosomal membrane to be degraded inside the lysosome. (b) Macroautophagy. Cytosolic material is sequestered by an expanding membrane sac (phagophore) forming a double-membrane vesicle, an autophagosome. Fusion of the autophagosome to the lysosome will expose the content of the autophagosome to lysosomal hydrolases. (c) Microautophagy. Small proteins can be engulfed directly by the lysosome without intermediate vesicles.

Based on how the proteins reach the lysosome, autophagy can be classified as (i) macroautophagy, (ii) microautophagy and (iii) chaperone-mediated autophagy CMA (Figure 1) [11].

- Macroautophagy, usually identified simply as autophagy, is the strategy commonly used for bulk degradation of cytoplasmic proteins and organelles (including dysfunctional mitochondria, which sometimes are referred as mitophagy). It is generally considered to be a nonspecific process in organisms from yeast to humans (with exceptions) and is a multistep process, where the formation of the double-membrane autophagic vacuoles (AVs) or autophagosomes occurs first. These vesicles surround the organelles or proteins to be degraded [12] and later fuse with endosomes to form an intermediate type of vesicle (amphisomes), or directly to lysosomes (autophagolysosomes), where the content will be finally degraded [4]. Macroautophagy can also be induced under conditions of physiological stress, like starvation [13]. The proteins regulating the whole process are autophagy-related proteins (Atg in yeast, ATG in mammals) which were discovered in yeast and have been found highly conserved. Up to date, more than 30 Atg proteins in mammals are known to participate in this intricate process (for review [11]).
- Microautophagy is a much simpler process and occurs when lysosomes engulf cytosolic components directly by membrane involution [14, 15].
- Finally, chaperone-mediated autophagy (CMA) incorporates cytosolic proteins that are brought by chaperones to the lysosome membrane (for review [16]). All the CMA substrates described so far are soluble cytosolic proteins containing a consensus sequence Lys-Phe-Glu-Arg-Gln (KFERQ) [17]. This motif (present in approximately 30% of cytosolic proteins) is recognized by a cytosolic chaperone, heat-shock cognate protein 70 (Hsc70), which transfers protein substrates to the lysosomal membrane, and there, through binding to the receptor lysosome-associated membrane protein-2A (LAMP-2A), they are translocated into the lysosome.

### 2.1. Autophagy versus Proteasome-Mediated Protein Degradation

Proteasomes are barrel-shaped protein complexes that mainly degrade small, short-lived nuclear and cytosolic proteins [18]. The ubiquitin-proteasome system is also important for the degradation of misfolded proteins in the endoplasmic reticulum [19]. Most proteins are targeted for proteasomal degradation after being covalently modified with ubiquitin. However, substrates need to be unfolded to pass through the narrow pore of the proteasome barrel, which hinders the clearance of oligomeric and aggregated proteins [20]. Under normal circumstances, the ubiquitin-proteasome system is more efficient than basal levels of macroautophagy, so for proteins that have access to both pathways, proteasomes are the favored clearance route. However, when a cytosolic protein is susceptible of aggregation, and therefore a poor proteasome substrate, macroautophagy will become the dominant clearance route [21]. This suggests that dependence of proteins on the macroautophagy pathway for their clearance correlates with their propensity to aggregate [22]. On the other hand, impairment of proteasome pathways has been associated with PD [23, 24] and HD [25]. Moreover, systemic exposure to proteasome inhibitors induces a model of Parkinson [23]. Therefore, it is not surprising that one of the most studied genes associated with familial PD was parkin, which encodes for an Ubiquitin-protein ligase [26]. However, whether proteasomal impairment is a key stepin familial or sporadic PD in which there are no primary defects in the ubiquitin-proteasome pathway is still not clear.

## 3. Proteasome and Protein Aggregates: Macroautophagy

Maybe as a consequence of proteasome impairment, or other reasons in familial forms of PD, aggregates of *α*-synuclein forming the characteristic Lewy bodies (LB) are found. Furthermore, mutant forms of *α*-synuclein are strongly dependent on the macroautophagy pathway [22, 27]. Confirming these findings, it has been shown that inhibition of macroautophagy has much smaller effects (if any effect at all) on the clearance of wild-type *α*-synuclein than on the clearance of the mutant aggregate-prone species [27]. This is also the case for other aggregates such as Htt in HD [28, 29]. 

Beclin-1 (a mammalian homologue of ATG6) is required for the formation of the autophagosome; alterations in beclin-1 have been linked to PD. Mutations in the PTEN-induced putative kinase 1 (PINK1) gene also cause autosomal recessive PD. The full-length PINK1 interacts with Beclin1, and the overexpression of PINK1 significantly enhances both basal and starvation-induced autophagy, which can be reduced by beclin1 gene knockdown. On the other hand, when a lentivirus expressing beclin-1 was delivered to the brain of *α*-synuclein transgenic mouse, enhanced lysosomal activation and reduction of accumulation of *α*-synuclein were observed [30]. However, overexpression of both mutant and wild type *α*-synuclein may also be accompanied by the induction of macroautophagy [31]. Moreover, functional deficiency of DJ-1 (associated with familiar forms of PD), and mutant forms of LRRK2 (leucine-rich repeat kinase 2, also linked to PD), lead to increased autophagy in murine and human cells [32] and in transfected cells [33]. 

Although neuronal autophagy appears primarily to be a protective process in the nervous system, it can also play a paradoxical role in neuronal death. With respect to the role of autophagy in neuronal death, several studies employing PD toxins, a mutant familial PD gene, and postmortem PD brains have demonstrated an important role for autophagy in promoting the death of dopamine neurons. For example, autophagic cell death has been observed in nigral dopamine neurons of PD patients [34]. MPP+ or dopamine toxicity-induced oxidative stress increases the number of AVs, autophagy, and cell death, all of which differs from what is observed in starvation-induced autophagy [35]. These studies suggest that pathogenic autophagy associated with neuronal death occurs and may be distinct from basal neuronal autophagy. The contribution of autophagy and autophagic cell death to degeneration of dopamine neurons may vary depending on the initial cause and specific cellular context [36]. A better understanding of autophagic stress and further identification of autophagic cell death mechanisms may lead to therapeutics that help restore homeostasis to dopamine neurons in PD.

## 4. Chaperone-Mediated Autophagy

Several of the 10 genes known to be mutated in association with PD encode proteins with sequences compatible with the CMA-targeting motif [16]. *α*-synuclein is degraded by macroautophagy (as discussed earlier) but also by CMA [27, 37]. Interestingly, it has been reported that mutant *α*-synuclein cannot be degraded by CMA, but, in addition, it seems to act as a blocker for other proteins using this pathway. Moreover, in sporadic Parkinson, where no mutations of *α*-synuclein are found, dopamine adducts of *α*-synuclein [38] behaved like the mutant protein, that is inhibiting cellular CMA process [39]. Other proteins like the myocyte-specific enhancer transcription factor 2D (MEF2D), that is, a *bona fide* CMA substrate [40], have been observed to increase their cytosolic levels in mice models of PD, in PD patients [41] and in neurons with partial blockage of CMA [40]. In these reports, blockage of CMA process seems to be a causal factor in the onset of PD. 

## 5. Mitocondria ROS/RNS and Autophagy

**Figure 2:**
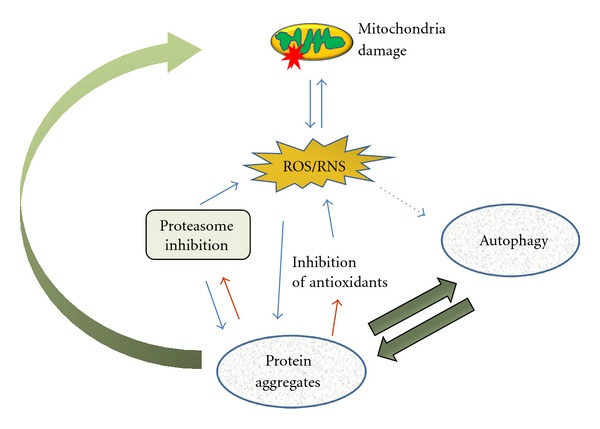
ROS/RNS production as a result of defective mitochondria respiratory activity can be induced by a number of factors like protein aggregates. Reactive species can also be generated by other cellular oxidases. These ROS/RNS species can modify several proteins which can stimulate and/or inhibit autophagy. In addition, reactive species produced or not in the mitochondria can target this organelle and induce further damage to it.

Mitochondrion is the major source of ATP in the cell; this energy is obtained via a multistep process where carbons atoms are oxidized to CO_2_. Damaged mitochondria are accumulated, and that contributes to inefficient oxygen reduction. As a result, highly reactive species both oxygen and nitrogen derived (ROS/RNS) are formed. Defective mitochondria are not the only source of ROS and RNS, but, regardless of their source, reactive species can in turn target mitochondria (Figure 2). Cells have efficient systems to detoxify ROS and RNS. When there is an excess of reactive species due to altered balance in the production and removal of ROS/RNS, pathological conditions such as PD and other neurodegenerative diseases associated occur [42–44]. It is generally accepted that autophagy is responsible for diminishing ROS/RNS damage, but, given the variety of reactive molecules and their location, it is yet not clear whether this is the case in every situation. Specific forms of ROS and RNS include hydrogen peroxide (H_2_O_2_), superoxide (O_2_^•−^), nitric oxide (NO), and peroxynitrite (ONOO^−^). Lipid peroxidation is a consistent feature of neurodegenerative diseases, and biologically active RLS, such as HNE (4-hydroxynonenal), accumulates in brains of patients with PD and AD [45–47].

The aggressiveness of these molecules makes them toxic to the cells; they react with proteins and lipids, inactivating them or making them prone to aggregation. For instance, *α*-synuclein and parkin have been found to be S-nitrosylated (addition of NO to thiol groups) in relation to PD [48]. Nitrogen modified *α*-synuclein makes the protein prone to aggregation [49, 50], and S-nitrosylation of parkin inactivates it [51]. It was recently shown that parkin is selectively recruited to damage mitochondria by PINK1, a mitochondrial serine/threonine kinase, and another recessive autosomal mutated gene linked to inherited forms of PD. PINK1 is usually present at low levels on the mitochondrial membrane [52]. When the mitochondrial membrane potential is dissipated, full-length PINK1 is accumulated in the outer mitochondrial membrane. Thus, damage to mitochondria facilitates the rapid accumulation of PINK1, and, subsequent to it, parkin is recruited to the mitochondria to induce mitophagy [53]. This discovery revealed a link between the mitochondrial quality control and proteins mutated in familial PD. Moreover, it further implicates a failure to eliminate dysfunctional mitochondria in the pathogenesis of PD. In addition, the voltage-dependent anion channel 1 (VDAC1) is a target for parkin-mediated polyubiquitination of Lys 27 and mitophagy [54]. Thus, pathogenic parkin mutations, together with PINK1 mutations, could lead to the disruption of mitochondrial recruitment of parkin, ubiquitination of mitochondrial substrates, formation of AVs, and the final clearance of damaged mitochondria *via *mitophagy. This putative role of PINK1 as a “guardian of mitochondrial integrity” seems to be confirmed by reports of PINK1 and parkin knockout models, where an accumulation of damaged mitochondria in various tissues including dopamine neurons occurs [55, 56]. Moreover, *α*-synuclein also targets to mitochondria, where it causes a decrease in complex I activity and/or mitochondrial damage [57, 58]. This mitochondrial damage causes an increase in mitophagy, presumably as an attempt to clear damaged mitochondria [59]. If mitophagy was not adequate for clearance of dysfunctional mitochondria, these deficiencies could contribute to cell death and neurodegeneration [60–62]. Altogether, these observations suggest that neuronal autophagy is essential for the turnover of damaged mitochondria and that the failure to induce mitophagy may underlie the selective dopaminergic neuronal loss observed in PD. This notion led to postulate that stimulation of mitophagy in dopaminergic neurons could serve as a therapeutic target to slow disease progression in PD. However, other reports show that PINK1 loss of function mutation, can also induce the opposite effect, induction of mitophagy [63]. On the other hand, mutations in LRRK2, an autosomal dominant gene involved in PD, have been shown to induce or inhibit autophagy depending on the cell type [33, 64].

## 6. Concluding Remarks

We have reviewed some of the mechanisms underlying gene mutations associated with autophagy in PD familial cases. Autophagy is a natural cell process to remove protein aggregates, dysfunctional mitochondria, and other potentially toxic proteins or organelles. Whether protein aggregates observed in neurodegenerative disorders are causal factors in the onset of disease is still an open debate. Consistent with this controversy, both deficits and stimulation of autophagy have been reported to underlie neurodegeneration. Thus, current scientific evidence shows that altered protein and organelle clearance, either by excess or deficit, are involved in the onset of PD. However, the mechanisms that could explain this apparently paradoxical behavior are not clear, and further investigation is required in order to use the autophagy machinery and mitochondria and protein-aggregates removal as an effective and safe therapeutic strategy in the treatment of familial and sporadic PD.
